# Validation of the SNACOR clinical scoring system after transarterial chemoembolisation in patients with hepatocellular carcinoma

**DOI:** 10.1186/s12885-018-4407-5

**Published:** 2018-04-27

**Authors:** Aline Mähringer-Kunz, Arndt Weinmann, Irene Schmidtmann, Sandra Koch, Sebastian Schotten, Daniel Pinto dos Santos, Michael Bernhard Pitton, Christoph Dueber, Peter Robert Galle, Roman Kloeckner

**Affiliations:** 1 Department of Diagnostic and Interventional Radiology, Johannes Gutenberg-University Medical Center Mainz, Langenbeckst. 1, 55131 Mainz, Germany; 2 Department of Internal Medicine, Johannes Gutenberg-University Medical Center Mainz, Mainz, Germany; 3 Clinical Registry Unit (CRU), Johannes Gutenberg-University Medical Center Mainz, Mainz, Germany; 4 Institute of Medical Biostatistics, Epidemiology and Informatics, Johannes Gutenberg-University Mainz, Mainz, Germany; 5 Department of Radiology, University Hospital Cologne, Cologne, Germany

**Keywords:** Hepatocellular carcinoma, Transarterial chemoembolisation, SNACOR

## Abstract

**Background:**

Transarterial chemoembolisation is the standard of care for intermediate stage (BCLC B) hepatocellular carcinoma, but it is challenging to decide when to repeat or stop treatment. Here we performed the first external validation of the SNACOR (tumour Size and Number, baseline Alpha-fetoprotein, Child-Pugh and Objective radiological Response) risk prediction model.

**Methods:**

A total of 1030 patients with hepatocellular carcinoma underwent transarterial chemoembolisation at our tertiary referral centre from January 2000 to December 2016. We determined the following variables that were needed to calculate the SNACOR at baseline: tumour size and number, alpha-fetoprotein level, Child-Pugh class, and objective radiological response after the first transarterial chemoembolisation. Overall survival, time-dependent area under receiver-operating characteristic curves, Harrell’s C-index, and the integrated Brier score were calculated to assess predictive ability. Finally, multivariate analysis was performed to identify independent predictors of survival.

**Results:**

The study included 268 patients. Low, intermediate, and high SNACOR scores predicted a median survival of 31.5, 19.9, and 9.2 months, respectively. The areas under the receiver-operating characteristic curve for overall survival were 0.641, 0.633, and 0.609 at 1, 3, and 6 years, respectively. Harrell’s C-index was 0.59, and the integrated Brier Score was 0.175. Independent predictors of survival included tumour size (*P* < 0.001), baseline alpha-fetoprotein level (*P* < 0.001) and Child-Pugh class (*P* < 0.004). Objective radiological response (*P* = 0.821) and tumour number (*P* = 0.127) were not additional independent predictors of survival.

**Conclusions:**

The SNACOR risk prediction model can be used to identify patients with a dismal prognosis after the first transarterial chemoembolisation who are unlikely to benefit from further transarterial chemoembolisation. However, Harrell’s C-index showed only moderate performance. Accordingly, this risk prediction model can only serve as one of several components used to make the decision about whether to repeat treatment.

## Background

Hepatocellular carcinoma (HCC) is one of the most common cancers worldwide and the second most common cause of cancer-related deaths [1, 2]. According to the Barcelona Clinic Liver Cancer (BCLC) classification, transarterial chemoembolisation (TACE) is the recommended treatment for intermediate-stage HCC (BCLC-B) [3]. However, the BCLC-B subgroup is quite heterogeneous, and not all patients benefit equally from TACE [4]. The question of when to stop TACE and possibly change to systemic treatment or even to best supportive care remains a challenge. In recent years, several scoring systems have been developed to support decision making after the first TACE, including the ART score (Assessment for Retreatment with TACE) and the ABCR score (Alpha-fetoprotein, BCLC, Child-Pugh, and Response) [5, 6]. However, none of these scoring systems are currently used in clinical practice.

To provide decision support regarding the issue of TACE retreatment, Kim et al. recently introduced the SNACOR (tumour Size, tumour Number, baseline Alpha-fetoprotein level, Child-Pugh class, and Objective radiological Response) clinical scoring system [7]. This system uses baseline liver function, baseline tumour parameters, and tumour response after the first TACE to evaluate the suitability of retreatment. However, the use of such clinical scoring systems in clinical routine has been controversial, and further external validation has been recommended [8, 9]. A few studies have been conducted to validate the ART score [10–14] and the ABCR score [13], but, to the best of our knowledge, no attempt has been made to validate the SNACOR score. Therefore, the purpose of this study was to perform the first external validation of the SNACOR score.

## Methods

### Patients

The study was approved by the institutional review board (IRB) for the retrospective analysis of clinical data. Patient records and clinical information were deidentified prior to analysis. Primary data collection was carried out using specially developed clinical registry software for the characterisation of patients with HCC [15].

The inclusion and exclusion criteria were the same as in the original SNACOR publication. The study included treatment-naïve patients who received TACE as first-line therapy and who had HCC diagnosed by histological or radiological evaluation according to the American Association for the Study of Liver Diseases (AASLD) or the European Association for the Study of the Liver (EASL) guidelines [7, 16, 17]. The study excluded patients with an inadequate target lesion (infiltrative pattern, non-arterial enhancement, or largest lesion < 1 cm); patients with an additional primary malignancy in another organ or with extrahepatic lesions; Child-Pugh class C patients; and patients with uncontrolled functional or metabolic disease [7].

As recommended by the authors of the original SNACOR publication, who only included patients who underwent conventional TACE, patients in this study received conventional, Lipiodol-based TACE (cTACE), or TACE using drug-eluting beads (DEB-TACE) [7]. Treatment was performed in a standardised manner that is extensively described elsewhere [18, 19].

### Imaging and tumour response

Each patient underwent contrast-enhanced computed tomography (CT) or magnetic resonance imaging (MRI) prior to the first TACE treatment. Six weeks after the first TACE treatment, restaging with CT or MRI was performed prior to the second TACE. This examination was the basis for the radiological assessment of the tumour response, which was evaluated by applying the unidimensional EASL criteria [20]. The objective tumour response was defined as a partial response (PR) before the second TACE treatment. Stable disease (SD) and progressive disease (PD) were assessed as a lack of radiological response.

### Calculation of the SNACOR score

The SNACOR score consists of the summed scores of the following variables: tumour size (< 5 cm, 0 points; ≥5 cm, 1 point), tumour number (< 4, 0 points; ≥4, 2 points), baseline alpha-fetoprotein level (< 400 ng/ml, 0 points; ≥400 ng/ml, 3 points), Child-Pugh class (A, 0 points; B, 1 point), and the objective radiological response (CR + PR, 0 points; SD + PD, 3 points). Hence, the SNACOR score ranges from 0 to 10 points. According to the original SNACOR paper, three risk groups can be differentiated using the SNACOR score: 0–2 points, low risk; 3–6 points, intermediate risk; and 7–10 points, high risk [7].

### Statistical analysis

Overall survival (OS) was defined as the period from the day before the first TACE until death or last follow-up. Kaplan-Meier survival curves were drawn using R 3.4.2 (A Language and Environment for Statistical Computing, R Foundation for Statistical Computing, https://www.R-project.org; accessed 2017). Survival between strata was compared using the log-rank test. Kernel probability densities were obtained using the R package survPresmooth, which calculates presmoothed probability density estimates for censored data [21]. Cumulative/dynamic receiver operating characteristic (ROC) curves were obtained using the R package timeROC. Areas under the curve (AUROCs) were derived at specified time points for comparison with those in the original SNACOR paper.

R 3.4.2 and SAS 9.4 were used for descriptive statistics and to perform multivariate analyses of all variables used in the SNACOR system in order to identify independent predictors of survival and to calculate hazard ratios (HRs) with corresponding 95% confidence intervals (CIs). As this analysis was intended to be exploratory, the *P*-values should be interpreted in a descriptive manner.

Validation was performed using Harrell’s C-index, and prediction error curves were based on the Brier score [22, 23]. Both Harrell’s C-index and AUROC can range from 0 to 1, where 0.5 indicates no predictive ability and 1 indicates perfect predictive ability. A value below 0.5 indicates “anti-prediction”. The Brier score at time t is the mean squared difference between the observed outcome (1 for event and 0 otherwise) and the predicted outcome probability at time t. The integrated Brier score (IBS) over the interval [0 m, 72 m] was calculated as a summary measure of prediction error.

## Results

### Patient recruitment

**Fig. 1:**
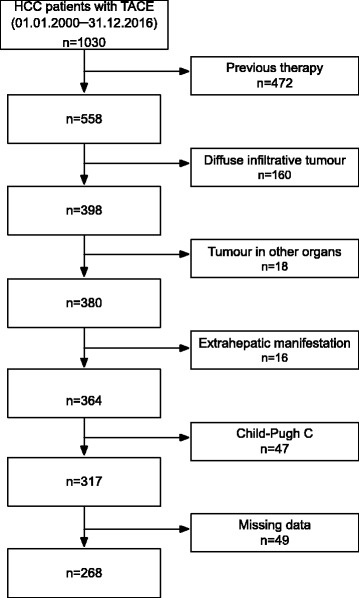
CONSORT flow diagram showing the reasons for drop-out and the final number of patients for whom the SNACOR score could be determined

A total of 1030 patients with HCC underwent TACE between January 2000 and December 2016 at our tertiary referral centre, and 762 patients were excluded for the reasons shown in the CONSORT flowchart (Fig. 1). Thus, the SNACOR score was calculated for 268 patients.

### Baseline patient characteristics and treatment

In our cohort, the mean patient age prior to the first TACE was 66.5 years (median, 66.9 years; range, 36.1–87.3 years; SD ± 9.4). A total of 227 (84.7%) patients were men, and 41 (15.3%) were women. The main aetiology of HCC was alcohol abuse. Table 1 shows the baseline patient characteristics of our cohort and those of the original SNACOR cohort. cTACE was performed in 190 patients, and DEB-TACE was performed in 78 patients. Overall, the mean number of TACE sessions was 5.6 (median, 5; min, 1; max, 21).

**Table 1:** Baseline characteristics of patients with hepatocellular carcinoma in this study and in the original SNACOR study [7]

		This study		Original SNACOR study	
		n = 268	%	*n* = 340	%
*Prior to first TACE*					
Age, y	Mean ± SD	66.5 ± 9.4		58	
	Range	36.1–87.3		51–65	
Sex	Male	227	84.7	274	80.6
	Female	41	15.3	66	19.4
Aetiology^a^	Alcohol	134	50.0		
	Hepatitis B virus	24	9.0	242	71.2
	Hepatitis C virus	77	28.7	44	12.9
	Other^b^	42	15.7	54	15.9
	No underlying liver disease	9	3.3	0	0
Child Pugh stage	A	184	68.7	288	84.7
	B	84	31.3	52	15.3
Tumour size, mm	Mean ± SD	52 ± 35		53	
	Range	10–215		27–88	
Number of nodes	1	78	29.1	127	37.4
	2	80	29.9	74	21.8
	3	44	16.4	31	9.1
	4	36	13.4	31	9.1
	≥5	30	11.2	77	22.6
Alpha–fetoprotein, ng/ml	Median	30.5		120.0	
	Range	0.5–920,910		17.1–1430.0	

^a^the sum of aetiologies is > 100% because patients could have two or more aetiologies

^b^“other” comprises: nonalcoholic steatohepatitis (*n* = 17; 6.3%), cryptogenic liver cirrhosis (*n* = 14; 5.2%), hemochromatosis (*n* = 11; 4.1%)

### SNACOR score

**Fig. 2:**
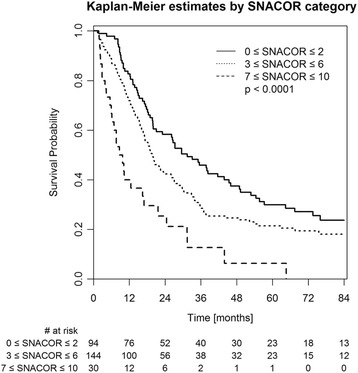
Kaplan-Meier survival curves according to SNACOR score category (*n* = 268) and log-rank test *p*-value

All variables that were needed to calculate the SNACOR score (both at baseline and prior to the second TACE) were determined (Table 1). Of the 268 patients, 94 (35.1%) were in the low-risk SNACOR score group (score 0–2), 144 patients (53.7%) were in the intermediate-risk group (score 3–6), and 30 patients (11.2%) were in the high-risk group (score 7–10). The median OS was 31.5 months (95% CI 23.1–46.0) in the low-risk group, 19.9 months (95% CI 17.1–26.2) in the intermediate-risk group, and 9.2 months in the high-risk group (95% CI 6.2–21.7). The Kaplan-Meier survival curves are shown in Fig. 2. Table 2 compares the survival rates in our study with those in the original SNACOR study [7].

**Table 2:** Comparison of the survival rates of patients with hepatocellular carcinoma in this study versus the survival rates of patients in the original SNACOR study

SNACOR, 3 subgroups	Low risk, 0–2 points	Intermediate risk, 3–6 points	High risk, 7–10 points	*P*-value
This study: median OS (95% CI), m	31.5 (23.1–46.0)	19.9 (17.1–26.2)	9.2 (6.2–21.7)	< 0.001
Original SNACOR study: median OS (95% CI), m	49.8 (34.3–65.3)	30.7 (25.8–35.6)	12.4 (5.9–18.9)	< 0.001

**Fig. 3:**
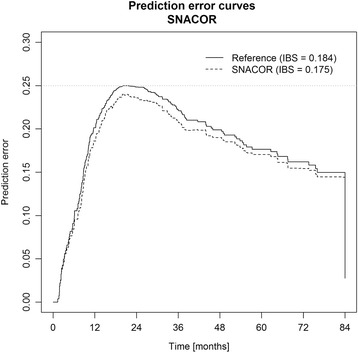
Prediction error curves and integrated Brier scores (IBS) for Kaplan Meier estimates based on the SNACOR score (SNACOR) and on the Kaplan Meier estimates for all patients without any stratification (reference)

**Fig. 4:**
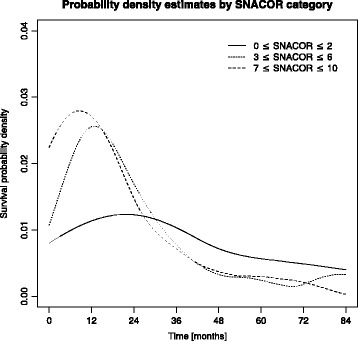
Presmoothed kernel estimates of the survival probability density according to SNACOR category (n = 268)

The AUROC for overall survival was 0.641 at 1 year, 0.633 at 3 years, and 0.609 at 6 years. Harrell’s C-index was 0.59. The prediction error curves are shown in Fig. 3. The IBS for the first 6 years was 0.175. In comparison, the IBS was 0.184 using the Kaplan-Meier estimates for the unstratified sample. The probability density estimates (Fig. 4) show a high degree of overlap.

The Cox regression analysis used tumour size, tumour number, baseline alpha-fetoprotein level, the Child-Pugh class, and objective radiological response as covariates (Table 3). Only tumour size, baseline alpha-fetoprotein level, and the Child-Pugh class had significant prognostic value (HR = 2.51, *P* < 0.001; HR = 1.76, *P* < 0.001; HR = 1.56, *P* = 0.004). Objective radiological response (HR = 0.97, *P* = 0.821) and tumour number (HR = 1.28, *P* = 0.127) were not additional independent predictors of survival.

**Table 3:** Proportional hazards model to identify independent predictors of survival and to compare hepatocellular carcinoma patient data in this study to the data of patients in the original SNACOR study [7]

	SNACOR parameters		Hazard ratio (95% CI)	*P*-value
This study	Tumour size	≥5 cm vs. < 5 cm	2.51 (1.88–3.36)	< 0.001
	Tumour number	≥4 vs. < 4	1.28 (0.93–1.75)	0.127
	Baseline AFP level	≥400 ng/ml vs. < 400 ng/ml	1.76 (1.28–2.43)	< 0.001
	Child-Pugh class	A vs. B	1.56 (1.16–2.12)	0.004
	Objective radiological response	CR + PR vs. SD + PD	0.97 (0.73–1.28)	0.821
Original SNACOR study	Tumour size	≥5 cm vs. < 5 cm	1.29 (0.95–0.17)	0.100
	Tumour number	≥4 vs. < 4	1.68 (1.24–2.28)	0.001
	Baseline AFP level	≥400 ng/ml vs. < 400 ng/ml	2.09 (1.55–2.82)	< 0.001
	Child-Pugh class	A vs. B	1.44 (0.96–2.14)	0.074
	Objective radiological response	CR + PR vs. SD + PD	2.24 (1.65–3.03)	< 0.001

## Discussion

In this study, the SNACOR score was able to differentiate between low-, intermediate-, and high-risk patients, who respectively showed a median OS of 31.5 months, 19.9 months, and 9.2 months. However, the original SNACOR publication reported respective median OS values of 49.8 months, 30.7 months, and 12.4 months for these groups. Hence, the discriminative ability of the SNACOR score between the three risk groups with respect to OS was inferior in our study compared to the original one. We observed considerable overlap in the survival time distribution. Accordingly, the Harrell’s C-index was 0.59 and the IBS was 0.175. AUROCs for overall survival were 0.641 at 1 year, 0.633 at 3 years, and 0.609 at 6 years; in the original SNACOR study, the comparable AUROC values were 0.756, 0.754, and 0.742, respectively. In summary, SNACOR does not perform well enough to be used alone to make clear-cut clinical decisions.

In the multivariate analysis, and in contrast to the original SNACOR study, we were only able to confirm the predictive value of tumour size, baseline alpha-fetoprotein level, and Child-Pugh class. Thus, two of the five parameters for calculating the SNACOR score were not predictive in our analysis, which may at least in part be due to the moderate sample size. The objective radiological response and tumour number at baseline failed to show a significant impact on survival. Notably, tumour size and tumour number reflect a patient’s tumour burden, and tumour size correlates with a higher risk of vascular invasion and distant metastasis [24, 25]. As tumour size is a known independent risk factor of survival [26, 27], it is part of several risk prediction models that have been published in recent years. We confirmed that tumour size is an independent predictor of survival. However, as noted above, tumour number was not an additional independent predictor of survival in our analysis. Whether or not tumour number is a significant prognostic factor is unclear in the literature; some series found it to have predictive value [27–30], while others did not [5, 26]. The fact that tumour number was not an independent predictor of survival in our study collective might be attributable to the moderate size of the final patient group of 268 patients. However, this validation group was considerably bigger than the validation cohort in the original SNACOR publication, which comprised 145 patients. Furthermore, it might be explained at least in part by the phenomenon of collinearity; we observed some positive correlation between tumour size and tumour number (Spearman *r* = 0.165). Alpha-fetoprotein level (AFP) was an independent predictor of survival in our analysis, which is in accordance with the majority of publications [27–29, 31], since AFP may be a surrogate marker for tumour burden and tumour aggressiveness [32, 33]. Therefore, AFP is part of several prediction scores [6, 26, 30]. The Child-Pugh score describes liver function and has shown significant prognostic value in several studies [28, 34–36]. Objective radiological response was not an additional independent predictor of survival in our analysis. Although it was not predictive in several other studies as well [10, 37], most authors regard objective radiological response as an important predictor [5, 6, 31, 38]. The fact that objective radiological response was not an independent predictor in our study might also be attributable to the moderate sample size and the phenomenon of collinearity, at least in part. We observed a weak negative correlation between tumour size and the objective radiological response (Spearman *r* = − 0.172). One important reason why the SNACOR score did not show the same predictive power in our study as in the original publication might be the so-called “overfitting” effect. This has been described as “a phenomenon occurring when a model maximizes its performance on some set of data but its predictive performance is not confirmed elsewhere due to random fluctuations of patients’ characteristics in different clinical and demographical backgrounds [8]”. Our patients differed significantly from the patients in the original SNACOR study in terms of tumour number, Child-Pugh class, and aetiology [7]. For example, alcoholic cirrhosis was the main reason for hepatocellular carcinoma in our study, whereas in the study by Kim et al., 71.2% of patients had hepatitis-B-related hepatocellular carcinoma, and 12.9% of patients had hepatitis-C-related hepatocellular carcinoma [7].

Our analysis has several limitations. The most important ones are that our validation was conducted in a retrospective manner and that the final sample size (*n* = 268) was only moderate. Ideally, prospective validation would be performed with a sufficiently large patient cohort using a multicentre approach. As recommended by the authors of the original SNACOR publication, which only included patients who underwent cTACE, in this study TACE was performed as cTACE or using DEB-TACE. Differences in TACE techniques might influence the applicability of the SNACOR system. cTACE and DEB-TACE have been compared multiple times in the last decade, but these comparisons have never shown a significant influence on survival [18, 39, 40]. Indeed, we drew the same conclusion when we analysed our own data [41]. Patients who underwent liver transplantation or surgery after TACE were excluded in the present analysis in order to ensure comparability with the original SNACOR data. However, from a statistical point of view, such patients should not be excluded; rather, they should be censored at the time of treatment change in order to eliminate immortal time bias.

## Conclusions

Even though the SNACOR system showed some ability to discriminate between patients with a favourable outcome after TACE versus patients with an impaired prognosis, SNACOR alone was not sufficient to reliably distinguish different prognostic groups. Therefore, SNACOR alone is not sufficient to support clear-cut clinical decision making, and further efforts are needed to determine appropriate criteria for making valid clinical predictions. Other approaches, such as machine learning, could be helpful for making future clinical predictions with increased validity.

## Abbreviations

AASLD: American association for the study of liver diseases
ABCR: Alpha-fetoprotein, BCLC, child-pugh, and response
AFP: Alpha-fetoprotein
ART: Assessment for retreatment with TACE
AUROC: Areas under receiver operating characteristic
BCLC: Barcelona clinic liver cancer classification
CI: Confidence interval
CT: Computed tomography
cTACE: Conventional transarterial chemoembolisation,
DEB-TACE: Drug-eluting bead transarterial chemoembolisation
EASL: European association for the study of the liver
HCC: Hepatocellular carcinoma
HR: Hazard ratio
IBS: Integrated brier score
IRB: Institutional review board
MRI: Magnetic resonance imaging
OS: Overall survival
PD: Progressive disease
PR: Partial response
ROC: Receiver operating characteristic
SD: Stable disease
SNACOR: Tumour size, tumour number, baseline alpha-fetoprotein level, child-pugh class, and objective radiological response
TACE: Transarterial chemoembolisation
